# Study on the spatiotemporal regulation of interferon-stimulated genes during Zika virus infection

**DOI:** 10.3389/fimmu.2025.1702266

**Published:** 2026-01-07

**Authors:** Xiao Zhong Chen, Yuan Xin Gong, Hong Xia Guo, Jiu Xiang He, TongYan Yang, Jin Tao Li

**Affiliations:** 1 Department of Biosafety, School of Basic Medicine, Army Medical University, Chongqing, China; 2 Chongqing Key Laboratory of Biosafety, Chongqing, China

**Keywords:** biomarkers, brain organoids, interferon signaling pathway, interferon-stimulated genes (ISGs), single-cell RNA sequencing, transcriptomics, Zika virus (ZIKV)

## Abstract

**Background:**

Zika virus (ZIKV) can cause severe neurological disorders such as congenital microcephaly, and there are currently no approved prevention or treatment measures. Elucidating the antiviral mechanism of the host interferon-stimulated genes (ISGs) is crucial for the development of therapeutic strategies.

**Methods:**

This study integrated multisystem transcriptomic data [human brain organoids, monocyte-derived dendritic cells (moDCs), and peripheral blood mononuclear cells (PBMCs) from infected patients], combined with cellular experiments and bioinformatics analysis, to systematically investigate the spatiotemporal regulation characteristics and functions of ISGs during ZIKV infection.

**Results:**

The results showed that treatment with interferon beta (IFN-β) significantly activated the ISGs (e.g., IRF7 and IFITM3) in the ZIKV-infected brain organoids, which were enriched in virus defense-related pathways. Single-cell transcriptomics revealed cellular heterogeneity in the IFN-β-driven ISG responses in moDCs. Transcriptomic analysis of the PBMCs identified 210 differentially expressed genes between the acute and convalescent phases. Core ISGs (e.g., RSAD2 and OAS3) were transiently upregulated in the acute phase. Through cross-analysis, 22 cross-system shared ISGs were screened out. Least absolute shrinkage and selection operator (LASSO) regression identified 11 potential diagnostic biomarkers for ZIKV infection. Small interfering RNA (siRNA) knockdown experiments confirmed that IFI35, IFI44, and OAS3 exerted antiviral effects by inhibiting ZIKV replication.

**Conclusion:**

This study reveals the functional plasticity of ISGs and provides key targets and theoretical support for the prevention and treatment of ZIKV.

## 1 Introduction

Zika virus (ZIKV), a single-stranded positive-sense RNA virus of the Flaviviridae family, was first isolated from rhesus monkeys in Uganda in 1947 (1). For decades after its discovery, ZIKV infections were only sporadically reported, as the majority of cases presented with self-limiting febrile illness and failed to attract widespread attention to its clinical harm. It was not until the 2015–2016 large-scale epidemic in Central and South America that ZIKV was first confirmed to induce severe neurological disorders (2), including Guillain–Barré syndrome (GBS) (3), congenital microcephaly, and meningoencephalitis—leading to renewed recognition of its public health threat (4). However, to date, there remain no approved therapeutic drugs or preventive vaccines for ZIKV, and breakthroughs in prevention and control measures are urgently needed (5).

Elucidation of the host–virus interactions—including the ZIKV replication mechanisms, the host factors involved, and the pathways of key host antiviral immune molecules—serves as the core scientific basis for the development of effective control strategies. In the early stage of viral infection, the host innate immune cells (e.g., dendritic cells and monocytes) rapidly activate the type I interferon (IFN-I) signaling pathway via pattern recognition receptors (PRRs)—mainly RIG-I-like receptors (RLRs) and Toll-like receptors (TLRs) (6). IFN-I further induces the expression of hundreds of interferon-stimulated genes (ISGs), which interfere with key processes such as viral entry, genome replication, protein translation, and progeny virion release, playing an irreplaceable core role in controlling viremia and blocking systemic viral spread (7). Current studies have identified several ISGs with definite anti-ZIKV activity, with typical examples including 2′,5′-oligoadenylate synthetase 1b (*OAS1B*) (8), interferon-stimulated exonuclease gene 20 (*ISG20*), interferon-induced transmembrane protein 3 (*IFITM3*) (9), *PML* genes (10), and interferon alpha-inducible protein 6 (*IFI6*) (11).

Notably, despite the key role of ISGs in restricting ZIKV infection, ZIKV has evolved multiple immune evasion mechanisms to antagonize the IFN-I signaling pathway. For instance, the ZIKV non-structural protein NS5 inhibits the IFN-I responses by directly binding to and mediating the degradation of signal transducer and activator of transcription 2 (STAT2) (12). Non-structural protein 3 (NS3) interferes with the RLR (RIG-I/MDA5)-mediated interferon (IFN) signaling by mimicking the binding motif of 14-3–3 proteins (13). These mechanisms suggest that ZIKV may exploit differences in the specific immune microenvironment of different host tissues to drive dynamic adaptive regulation of the ISG antiviral network, thereby achieving tissue colonization and persistent infection.

ZIKV exhibits broad host cell tropism. One of its core pathogenic features is the direct infection of neural progenitor cells (NPCs). By inducing cell cycle arrest and apoptosis, it ultimately causes severe neurological symptoms such as congenital microcephaly. At the same time, persistent viral presence in neural tissues triggers chronic inflammatory responses, further exacerbating neural damage. Previous studies have confirmed that exogenous IFN-I supplementation can alleviate ZIKV-induced neurotoxicity to a certain extent; however, the specific degree of functional conservation or divergence of the ISG network between the immune and nervous systems remains unclear.

Against the above research background, this study integrated bulk transcriptomics and single-cell transcriptomics to systematically explore the spatiotemporal regulation of ISGs during ZIKV infection, focusing on analyzing their dual functions in immune response and neuroprotection. The results not only reveal the previously unrecognized functional plasticity of ISG effectors but also provide key experimental evidence and theoretical support for the subsequent development of ZIKV infection therapeutic strategies targeting specific ISGs.

## 2 Materials and methods

### 2.1 Data sources and dataset information

All public transcriptomic datasets used in this study were retrieved from the Gene Expression Omnibus (GEO) database of the National Center for Biotechnology Information (NCBI). The GSE123816 dataset includes transcriptomic data of ZIKV-infected human brain organoids treated with IFN, which was used to explore dynamic changes in the gene expression in brain organoids under IFN regulation (14). The GSE230571 dataset comprises single-cell transcriptomic data of IFN-treated, ZIKV-infected human monocyte-derived dendritic cells (moDCs), which was used to investigate the effect of IFN on the transcriptome of ZIKV-infected cells at the single-cell level (15). The GSE129882 dataset contains transcriptomic data of peripheral blood samples from patients with acute ZIKV infection, which was used to analyze host gene transcriptional changes during the acute infection phase (16).

### 2.2 Differentially expressed gene analysis and data visualization

Based on bulk transcriptomic data from the GSE129882 and GSE123816 datasets, differentially expressed genes (DEGs) were screened using the R package edgeR (9), with a significance threshold set at an adjusted *p*-value <0.05. The R package ggplot2 was used to generate visualizations such as volcano plots and box plots, while the pheatmap package was employed to draw heatmaps of the expression of the DEGs to illustrate the characteristics of the gene expression differences.

For the screened DEGs, Gene Ontology (GO) functional annotation [including biological process (BP), cellular component (CC), and molecular function (MF)] and Kyoto Encyclopedia of Genes and Genomes (KEGG) pathway enrichment analysis were performed using the R package clusterProfiler (17), with a screening criterion of *p* < 0.05. The aim was to reveal the core biological functions and signaling pathways involved in the DEGs.

Venn diagrams and the online tool EVenn (18) were used for cross-analysis of the DEGs from the GSE129882 and GSE123816 datasets to identify shared DEGs between the two datasets. These shared DEGs were input into the STRING database (https://string-db.org/) to construct a protein–protein interaction (PPI) network using default parameters (minimum interaction score ≥0.4). The PPI network was visualized using Cytoscape 3.9 software (19), and the built-in maximal clique centrality (MCC) algorithm was used to calculate the node connectivity scores. Genes with the highest connectivity scores were defined as hub genes.

### 2.3 Single-cell transcriptomic data analysis

Based on the single-cell transcriptomic data of moDCs from the GSE230571 dataset, normalization analysis was performed using the R package Seurat (20). Low-quality cells with RNA counts >60,000 or <500 and unique gene counts <1,000 were filtered out. The “LogNormalize” method was used to normalize the gene expression matrix of the remaining cells (scaling factor set to 10,000). The “vst” (variance stabilizing transformation) method was used to screen 2,000 highly variable genes (HVGs) per sample. The cells were divided into 20 clusters using the FindClusters function (clustering resolution set to 0.2), and the AddModuleScore function was used to calculate the activity score of the ISGs in each cluster. The FindMarkers function built into Seurat was used to screen the DEGs between different cell clusters (|log_2_FC| >1) to reveal the cell subset-specific transcriptional characteristics of moDCs under IFN regulation.

### 2.4 Prediction of potential therapeutic drugs

The 22 most significant DEGs (the 22 genes with the smallest adjusted *p*-values) screened in this study were input into the Connectivity Map (CMap) database (https://clue.io/query). The parameters (e.g., gene list format and similarity threshold) were set in strict accordance with the official database instructions, and small-molecule drugs with potential therapeutic effects on ZIKV infection were predicted through gene expression signature matching.

### 2.5 Cell culture and viral infection

The human non-small cell lung cancer cell line A549 (purchased from the American Type Culture Collection, catalog no. CCL-185) was used. Cells were cultured in a constant-temperature incubator at 37°C with 5% CO_2_ using complete Ham’s F-12K medium. The complete medium was formulated as follows: Ham’s F-12K basal medium (catalog no. 21127022) supplemented with 10% fetal bovine serum (FBS; catalog no. A5256701) and 1× penicillin–streptomycin double antibody (catalog no. 15140122; all from Gibco, Waltham, MA, USA).

When A549 cells grew to the logarithmic phase (confluency 70%–80%), they were infected with ZIKV (PRVABC59 strain) at a multiplicity of infection (MOI) of 1 and cultured for another 24 h after infection.

### 2.6 Quantitative real-time PCR validation

At 24 h after ZIKV infection, A549 cells were collected and the total RNA extracted using the RNeasy Mini Kit (cat. no. 74104; Qiagen, Hilden, Germany). The RNA purity was evaluated using a NanoDrop 2000 spectrophotometer, with an *A*_260_/*A*_280_ absorbance ratio ranging from 1.8 to 2.0 deemed acceptable for subsequent experimental procedures. A total of 1,000 ng of qualified total RNA was used to synthesize complementary DNA (cDNA) using the PrimeScript™ RT kit (catalog no. RR047A; TaKaRa, Shiga, Japan) under the following reaction conditions: 37°C for 15 min and 85°C for 5 s.

Using the synthesized cDNA as a template, detection was performed on a real-time fluorescent quantitative PCR instrument (LightCycler 96; Roche, Basel, Switzerland) with SYBR Green fluorescent dye (catalog no. RR420A; TaKaRa, Shiga, Japan). Glyceraldehyde-3-phosphate dehydrogenase (*GAPDH*) served as the internal reference gene to normalize for variations in the cDNA input and reaction efficiency. The relative fold change in the expression levels of target ISGs was calculated using the 2^−ΔΔ^*^C^*^t^ method. The nucleotide sequences of the primers employed in this assay are provided in **Supplementary Table S1**.

### 2.7 Transfection of siRNA into A549 cells

Small interfering RNA (siRNA) was transiently transfected into A549 cells using the Lipofectamine 3000 reagent (Invitrogen, Carlsbad, CA, USA). Approximately 8 × 10^4^ cells were seeded in each well of a 24-well plate. The next day, siRNA was transfected according to the kit instructions, with 40 pmol siRNA per well. At 48 h after transfection, the cells were infected with ZIKV. The siRNA was synthesized by Beijing Qikexin Technology Co., Ltd. (Beijing, China). Cytotoxicity was assessed by measuring the ATP levels with the CellTiter-Glo assay in accordance with the manufacturer’s instructions (catalog no. G7571; Promega, Madison, WI, USA).

### 2.8 Statistical analysis

All experimental data were independently repeated at least three times, and the measurement data were expressed as the mean ± standard deviation (mean ± SD). GraphPad Prism 9 software was used for statistical analysis: Student’s *t*-test was used for comparisons between two groups, and one-way analysis of variance (ANOVA) was used for comparisons among multiple groups. A *p*-value <0.05 was considered statistically significant.

## 3 Results

### 3.1 Transcriptomic identification of IFN-β-induced ISGs protecting against ZIKV-induced neural injury based on the human brain organoid model

Previous studies have confirmed that interferon beta (IFN-β) can alleviate the developmental defects of human brain organoids induced by ZIKV infection by regulating the transcriptional expression of downstream ISGs (14); however, the specific key effector ISGs mediating this neuroprotective function remain unclear. To identify the key effector ISGs of IFN-β in anti-ZIKV neuroprotection, this study used the RNA sequencing data of ZIKV-infected human brain organoid models (GSE123816 dataset) to systematically compare the expression characteristics of the ISGs between the IFN-β-treated and untreated groups.

**Figure 1:**
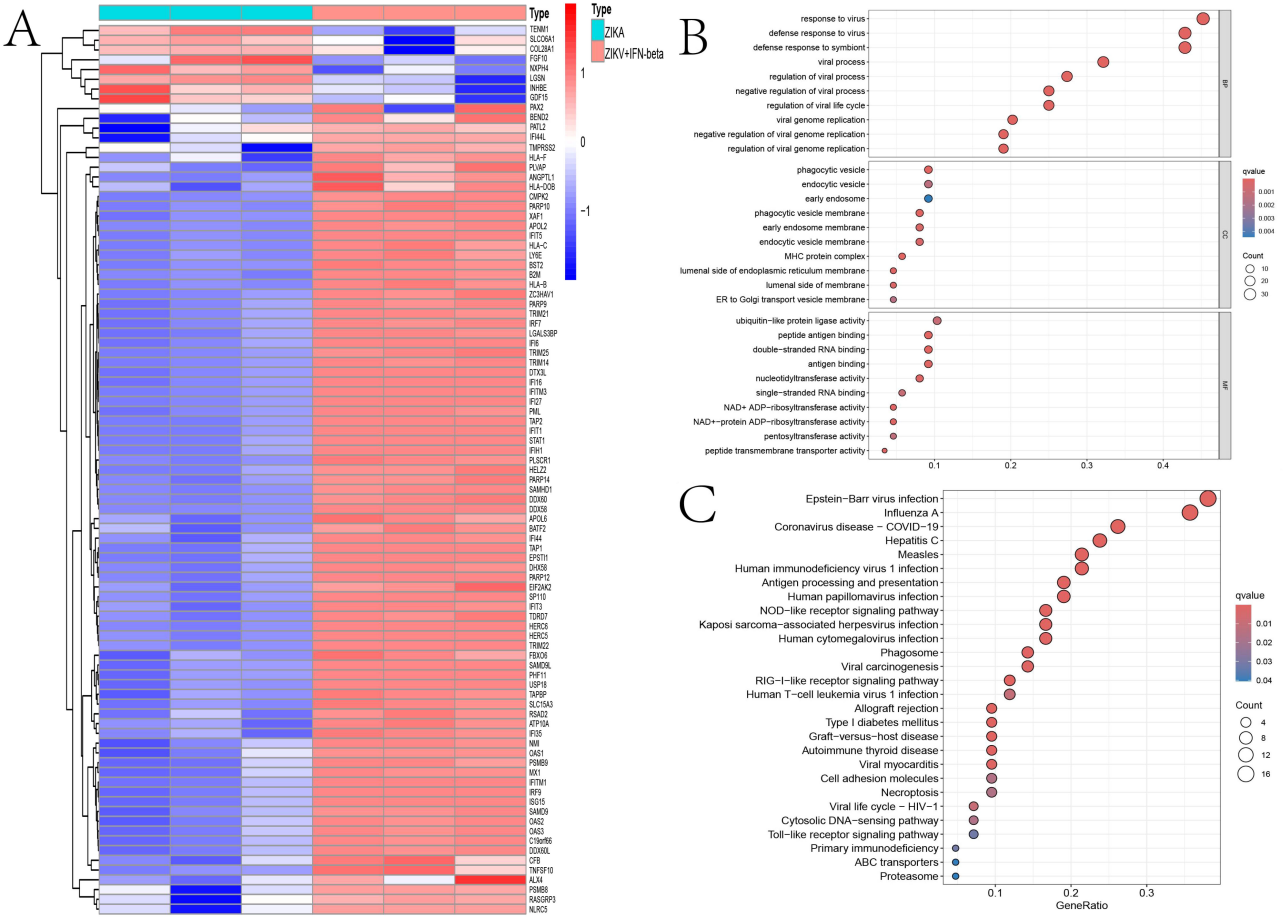
Transcriptomic features of Zika virus (ZIKV)-infected human brain organoids treated with interferon beta (IFN-β). **(A)** Heatmap of the differentially expressed genes (DEGs) in ZIKV-infected human brain organoids. **(B)** Bubble plots of the Gene Ontology (GO) enrichment analysis for the DEGs in IFN-β-treated *vs*. untreated ZIKV-infected human brain organoids. *MF*, molecular function; *BP*, biological process; *CC*, cellular component. **(C)** Kyoto Encyclopedia of Genes and Genomes (KEGG) pathway enrichment analysis of the DEGs in IFN-β-treated *vs*. untreated ZIKV-infected human brain organoids.

Using differential expression analysis [false discovery rate (FDR) < 0.05, |log_2_FC| > 1.5], we identified 91 DEGs in the IFN-β-treated ZIKV-infected organoids compared with the untreated group. Among these, 83 genes were upregulated and eight were downregulated (**Figure 1A**). Notably, the upregulated DEGs were enriched with members of ISG families with known antiviral functions, such as *IRF7*, *IRF9*, *IFI6*, and *IFITM3*, suggesting that these genes may play a core role in inhibiting ZIKV replication and in exerting neuroprotective effects.

GO and KEGG analyses of the 91 DEGs showed that they are mainly involved in biological processes including antigen processing and presentation, the RIG-I-like receptor signaling pathway, and the Toll-like receptor signaling pathway. These results further highlight the key role of IFN-β in the regulation of the response to ZIKV infection (**Figures 1B, C**).

### 3.2 Single-cell transcriptomics reveals the heterogeneity of IFN-β-driven antiviral ISGs in moDCs

Human moDCs are one of the main target cells of ZIKV. Due to their ability to mimic the host’s immune response to ZIKV infection *in vivo*, they have been widely used as an *in vitro* cell model to study ZIKV–host interactions (21). To systematically analyze the cellular heterogeneity of the IFN-β-driven ISG responses in moDCs, this study used single-cell transcriptome sequencing to analyze ZIKV-infected human moDC samples (dataset GSE230571). The experimental design included an IFN-β pretreatment group and an untreated control group to clarify the regulatory effect of IFN-β on the expression heterogeneity of the ISGs in moDCs.

**Figure 2:**
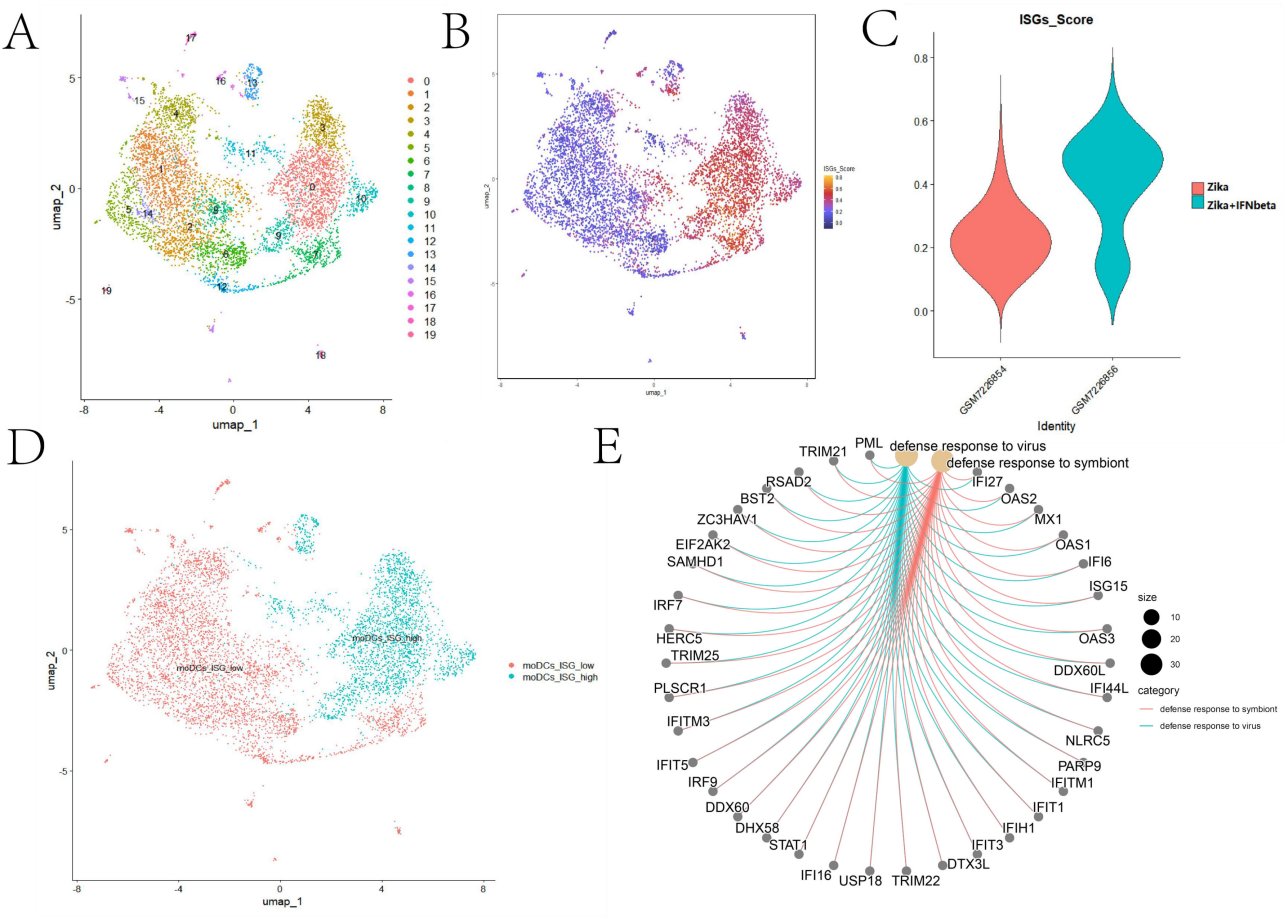
Single-cell transcriptomic features of Zika virus (ZIKV)-infected monocyte-derived dendritic cells (moDCs) treated with interferon beta (IFN-β). **(A)** Uniform manifold approximation and projection (UMAP) visualization of the moDCs. **(B)** UMAP plot of the moDCs overlaid with the interferon-stimulated gene (ISG) scores. **(C)** Violin plot comparing the ISG scores of the moDCs with and without IFN-β treatment. **(D)** UMAP plot of the moDCs highlighting the moDCs_ISG_low and moDCs_ISG_high clusters. **(E)** Circos plots showing the Gene Ontology (GO) enrichment analysis of the differentially expressed genes (DEGs) between the moDCs_ISG_low and moDCs_ISG_high clusters.

After strict sample quality control, uniform manifold approximation and projection (UMAP) dimensionality reduction analysis was performed to divide all qualified cells into 20 distinct clusters, which were visualized (**Figure 2A**). The AddModuleScore function in the Seurat package was used to calculate the ISG activity score of each cell cluster. The results (**Figures 2B, C**) showed that, compared with the untreated control group, all cell clusters of moDCs in the IFN-β pretreatment group exhibited higher ISG activity. This suggests that pretreatment with IFN-β can systematically activate the antiviral response program in moDCs, and this activation effect has broad coverage in the cell population.

To further clarify the functional differences of the ISGs in ZIKV-infected moDCs and their association with cellular heterogeneity, the 20 cell clusters were classified into two functional clusters based on their ISG activity scores: the ISG high-activity cluster (ISG_high) and the ISG low-activity cluster (ISG_low) (**Figure 2D**). The FindMarkers function in the Seurat package was used to analyze the DEGs between the two clusters, which enabled identifying 241 upregulated and 44 downregulated genes. The results showed that the ISG with the most significant expression difference between the two clusters was *IFI27* (interferon alpha-inducible protein 27). This result is consistent with previous studies concluding that *IFI27* plays a key role in IFN-mediated anti-dengue virus responses (22).

GO functional enrichment analysis of the upregulated genes between the ISG_high and ISG_low clusters (**Figure 2E**) showed that these DEGs were mainly enriched in biological processes such as “response to virus,” “defense response to virus,” and “negative regulation of viral process.” This further confirms that the ISG activity difference is a core functional heterogeneity feature of moDCs in response to ZIKV infection and that IFN-β can shape the heterogeneity of the moDC antiviral immune responses by regulating the ISG activity in different cell clusters.

### 3.3 Transcriptome of PBMCs from ZIKV-infected patients reveals dynamic regulation of ISGs

To systematically analyze the dynamic regulation of ISGs during natural ZIKV infection, this study used transcriptomics to compare the peripheral blood mononuclear cells (PBMCs) from ZIKV-infected patients in the acute and convalescent phases based on the GSE129882 dataset.

**Figure 3:**
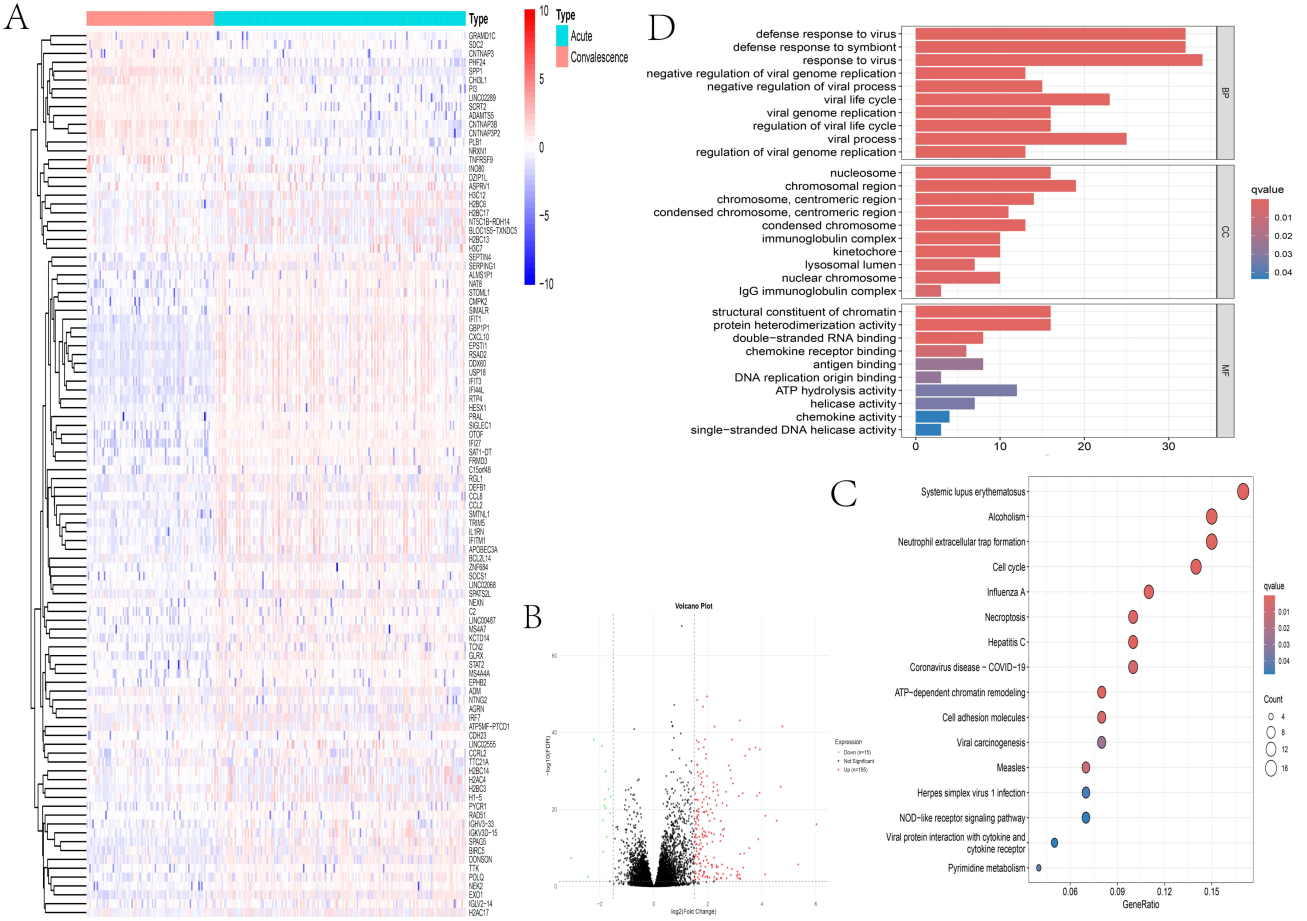
Transcriptional profiles of naturally acquired Zika virus (ZIKV) infection: acute *versus* convalescent phase. **(A)** Heatmap of the 50 differentially expressed genes (DEGs) in the peripheral blood mononuclear cells (PBMCs) from ZIKV-infected patients. **(B)** Volcano plot of gene expression (*red*: upregulated DEGs; *green*: downregulated DEGs; *black*: non-DEGs; |log_2_FC| > 1.5, FDR < 0.05). **(C)** Gene Ontology (GO) enrichment analysis of the DEGs. **(D)** Kyoto Encyclopedia of Genes and Genomes (KEGG) pathway enrichment analysis of the DEGs.

The DEGs were screened using the R package edgeR with thresholds set as FDR < 0.05 and absolute log_2_FC| >1.5. A total of 210 significant DEGs were identified in the acute phase compared with the convalescent phase: 195 were upregulated and 15 were downregulated. The core upregulated DEGs included typical ISGs such as *IRF7* and *IFITM1*, suggesting that the body establishes an antiviral defense by upregulating ISGs in the acute phase (**Figures 3A, B**).

The 210 DEGs were subjected to GO annotation and KEGG enrichment analysis using the R package clusterProfiler. For GO annotation, BPs were enriched in immune-related processes such as “response to virus” (**Figure 3C**), CCs were enriched in terms such as “chromosomal region,” and MFs were enriched in “chromatin structure constituent” and other terms. KEGG analysis revealed that pathways including “influenza A infection” were enriched (**Figure 3D**), indicating that the regulation of ISGs by the host is relatively conserved among different pathogens.

### 3.4 Identification of common ISGs in the nervous and immune systems during ZIKV infection

**Figure 4:**
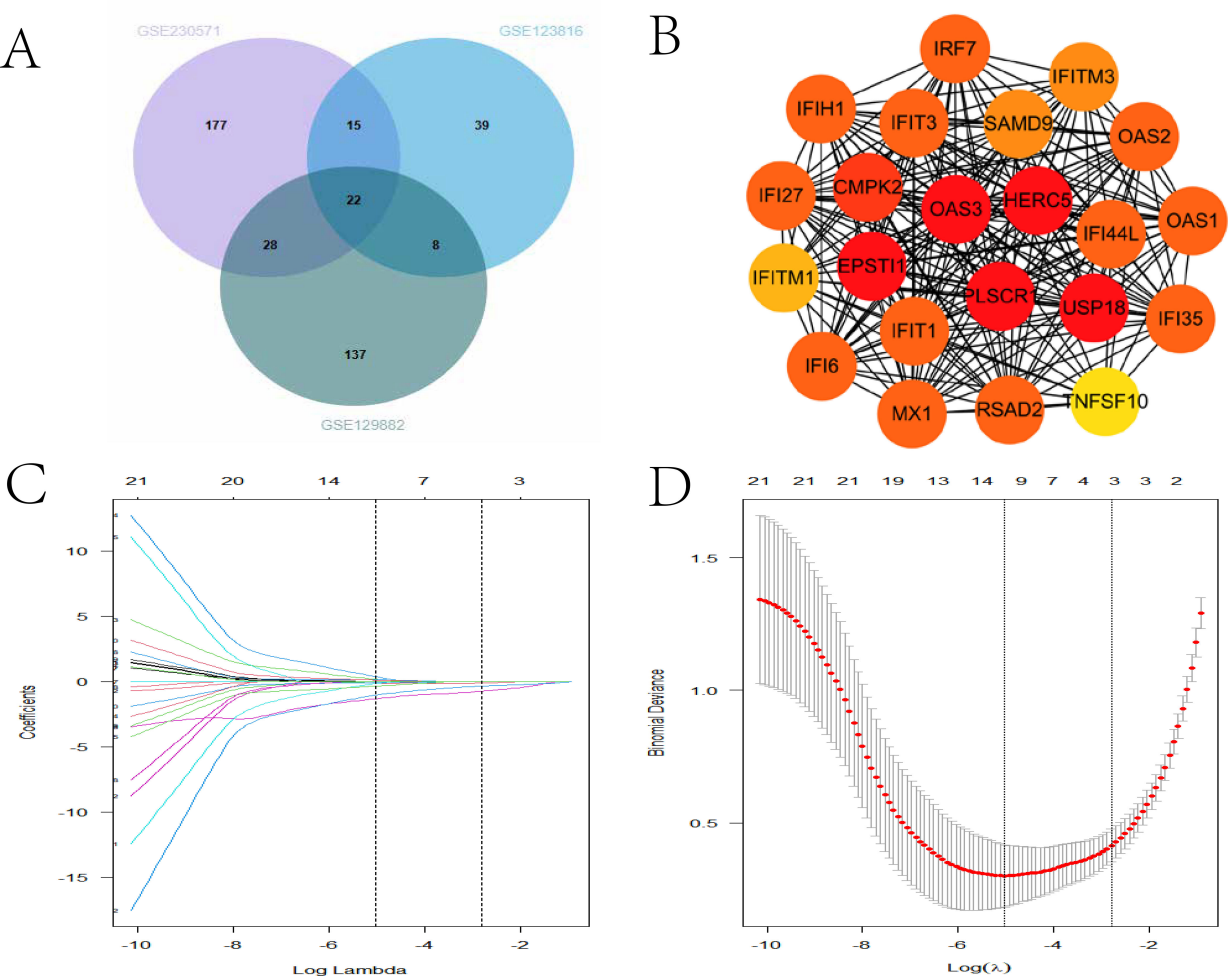
Screening of the key interferon-stimulated genes (ISGs) in Zika virus (ZIKV) infection. **(A)** Venn diagram showing the number of common differentially expressed ISGs across different datasets. **(B)** Protein–protein interaction (PPI) network of the 22 common differentially expressed ISGs identified from different datasets. **(C)** Tenfold cross-validation for tuning parameter selection in the least absolute shrinkage and selection operator (LASSO) regression model. **(D)** Partial likelihood deviance curve illustrating the selection of the minimum number of signature genes.

To identify ISGs that regulate ZIKV infection across both the nervous and immune systems—two key compartments targeted by ZIKV *in vivo*—we integrated two transcriptomic datasets that respectively represent these physiological systems. The first dataset was derived from ZIKV-infected human brain organoids (a validated *in vitro* model of the nervous system), while the second dataset was obtained from ZIKV-exposed PBMCs and moDCs—cell types that are critical for mediating antiviral immunity in the immune system. Cross-analysis was performed using the R package VennDiagram, which revealed 22 common DEGs among the DEGs of the two systems (**Figure 4A**). Interestingly, all of these DEGs are ISGs. Functional enrichment analysis confirmed that these common ISGs were significantly enriched in pathways related to viral infection response and immune regulation, suggesting that they may be key molecules mediating antiviral responses across systems. Furthermore, the STRING database was used to construct a PPI network of the 22 common ISGs, clarifying the pattern of molecular interactions (**Figure 4B**). At the same time, the CMap database was used to predict potential compounds that may regulate these genes, with detailed prediction results shown in **Table 1**.

**Table 1:** Compounds identified by connectivity map (CMap) analysis.

ID	Compound	raw_cs	fdr_q_nlog10	norm_cs
1	Clocortolone pivalate	0.987	4.4615	2.1849
2	BRD-A09719808	0.9853	4.3974	2.1811
3	BRD-K96704748	0.9838	4.3447	2.1777
4	QS-11	0.9825	4.3062	2.1748
5	BRD-K65342895	0.9816	4.2813	2.1729
6	BRD-K98039984	0.9813	4.2734	2.1722
7	Ispinesib	0.9812	4.2708	2.172
8	Suloctidil	0.9793	4.2237	2.1679
9	BRD-K90849765	0.9781	4.1962	2.1652
10	BS-181	0.9777	4.1869	2.1643
11	Berbamine	0.9771	4.1742	2.163
12	BRD-K74623475	0.9771	4.174	2.163
13	BRD-K93215584	0.9766	4.1625	2.1617
14	KU-60019	0.9762	4.1553	2.161
15	BRD-K94991378	0.9745	4.1225	2.1573
16	BRD-K55536701	0.9739	4.1103	2.1558
17	BRD-K41925105	0.9729	4.0925	2.1536
18	COL-3	0.9724	4.0838	2.1525
19	Pinaverium	0.9711	4.0623	2.1497
20	BRD-K41723848	0.971	4.0611	2.1495

To assess whether these 22 ISGs can be used as biomarkers for diagnosing ZIKV infection, a least absolute shrinkage and selection operator (LASSO) logistic regression model was employed for screening (**Figures 4C, D**), which ultimately identified 11 genes (i.e., *IRF7*, *IFIT3*, *IFI35*, *IFI44*, *IFIH1*, *IFI27*, *IFITM1*, *TNFSF10*, *RSAD2*, *OAS3*, and *EPSTI1*) as potential diagnostic biomarkers for ZIKV infection.

**Figure 5:**
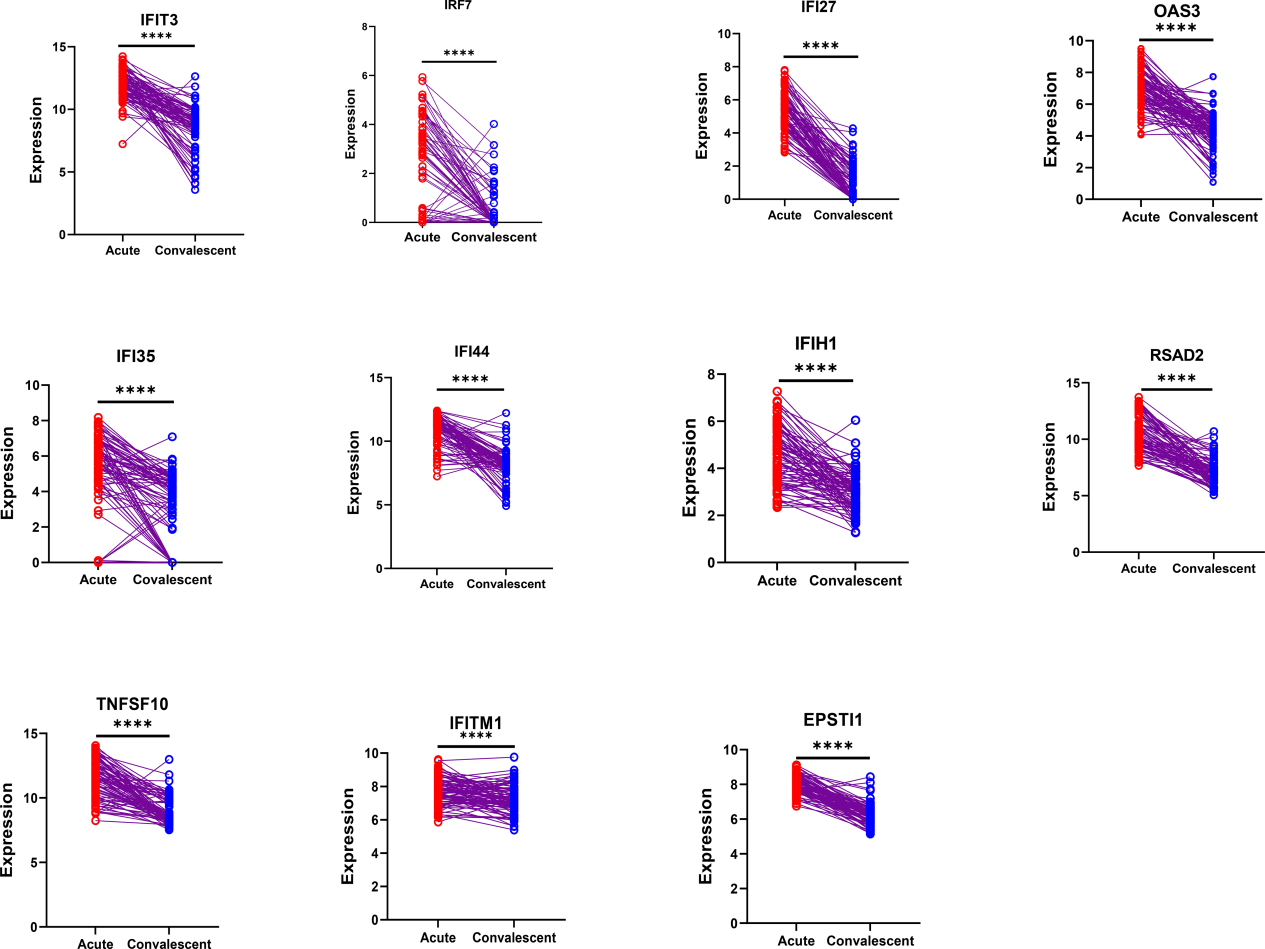
Expression analysis of the 11 signature genes in the peripheral blood mononuclear cells of Zika patients at different stages. *Asterisks* indicate significant differences. ****p<0.0001.

**Figure 6:**
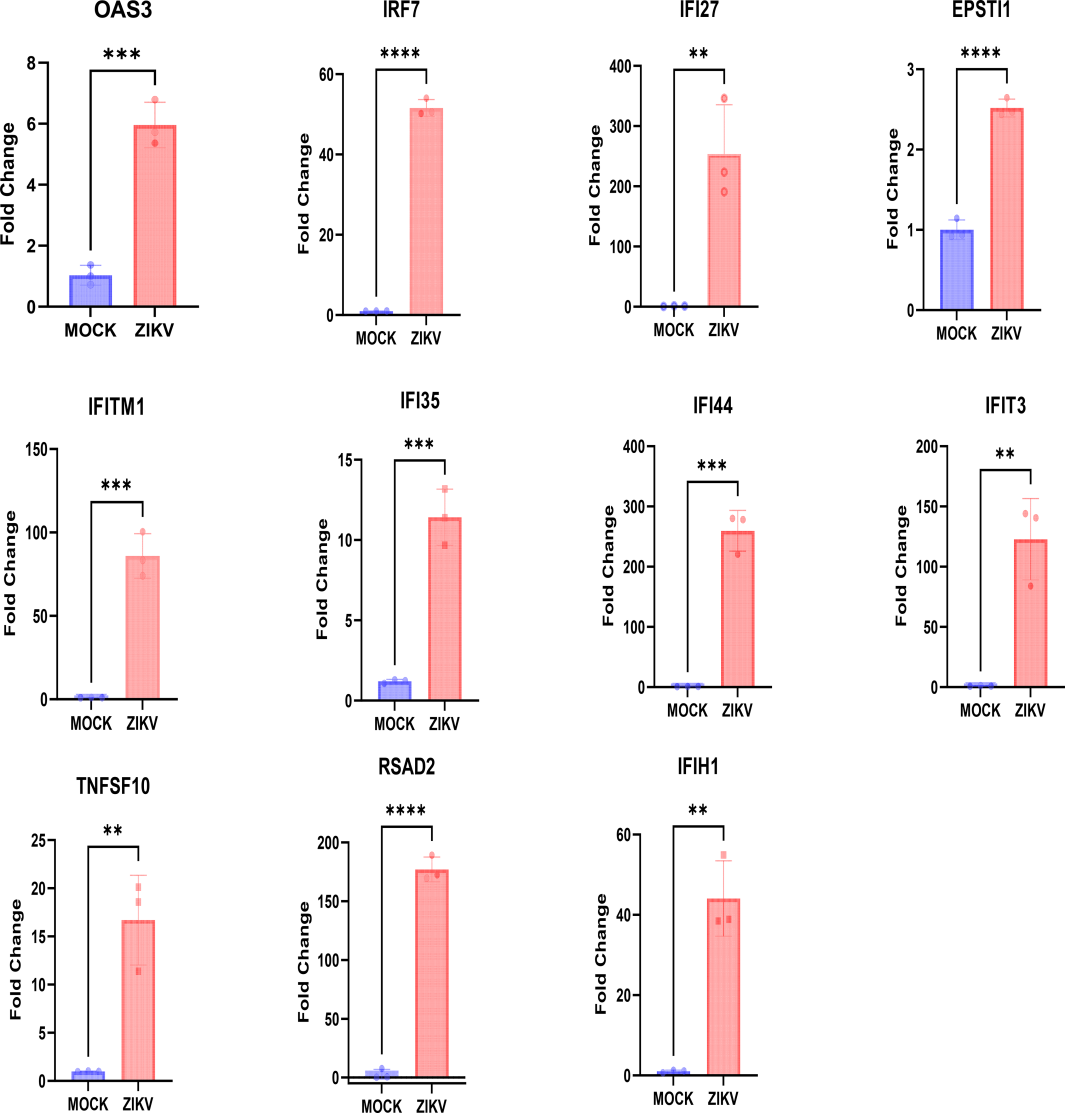
Fold change in the expression of the 11 interferon-stimulated genes (ISGs) in A549 cells after Zika virus (ZIKV) infection. Fold change in the expression of the 11 genes in A549 cells was determined 24 h after mock infection or ZIKV infection [multiplicity of infection (MOI) = 1] using RT-PCR. Gene expression was normalized to *GAPDH* and calculated using the 2^−ΔΔ^*^C^*^t^ method. Data are presented as the mean ± SD from at least three independent experiments. Statistical significance was assessed using a two-sided **p < 0.01; ***p < 0.001;****p < 0.0001.

These genes were significantly upregulated during the acute phase compared with the convalescent phase (**Figure 5**). To further clarify the expression characteristics of these 11 genes during ZIKV infection, human non-small cell lung cancer A549 cells were used as a model, and their expression levels were detected 24 h after ZIKV infection. Quantitative real-time PCR (qRT-PCR) was used to compare the relative expression levels of the 11 genes between the infected group and the mock-infected control group. The results showed that all genes were significantly upregulated in the infected group, with *IFI44* and *IFI27* showing the most significant upregulation (**Figure 6**), indicating that these genes may be involved in the regulation of the host’s response to ZIKV infection in the nervous and immune systems.

**Figure 7:**
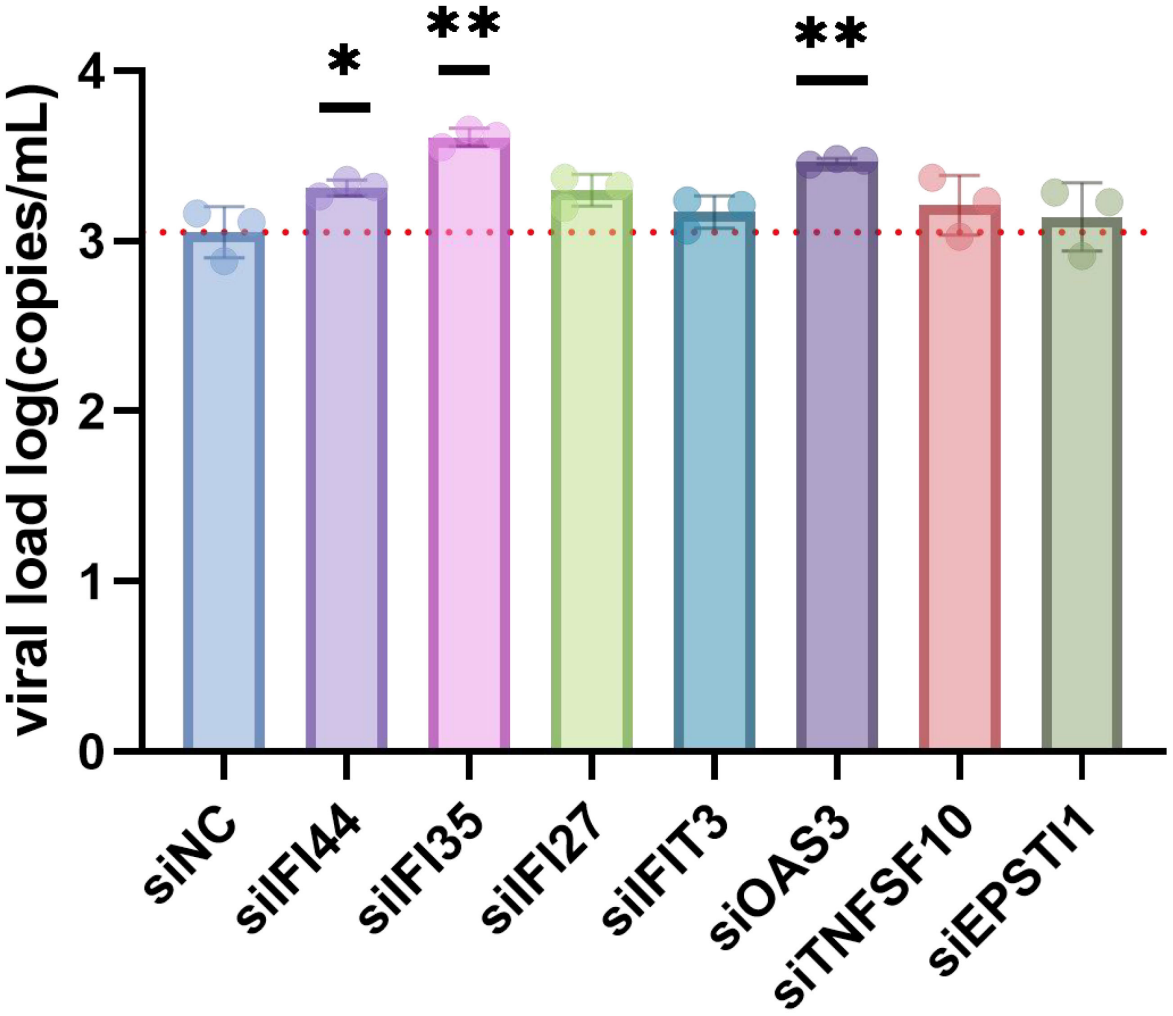
Effects of seven interferon-stimulated gene (ISG) knockdown on Zika virus (ZIKV) replication in A549 cells. A549 cells were transfected with small interfering RNA (siRNA) targeting candidate ISGs or non-targeting control siRNA for 48 h, followed by infection with ZIKV at a multiplicity of infection (MOI) of 1. The viral copy number in the supernatant was measured by quantitative real-time PCR (qRT-PCR) at 24 h post-infection. Data represent the mean ± SD from three or more independent experiments. Significance was determined using two-sided Student’s *t*-test. *p < 0.05; **p < 0.005.

Among the 11 candidate biomarkers, seven genes (i.e., *IFI44*, *IFI35*, *IFI27*, *IFIT3*, *OAS3*, *TNFSF10*, and *EPSTI1*) with no previous reports of association with ZIKV infection were selected for functional verification via siRNA-mediated gene knockdown. A549 cells were transfected with siRNAs targeting these genes. Subsequently, the transfected cells were infected with ZIKV and the viral RNA copy numbers determined. Knockdown of *IFI35*, *IFI44*, or *OAS3* resulted in a significant increase in ZIKV RNA copies compared with the non-targeting control. This confirms that these genes play an antiviral role in inhibiting ZIKV replication (**Figure 7**). To exclude the possibility that the enhanced viral replication resulted from siRNA-induced cytotoxicity, cell viability was assessed using the CellTiter-Glo assay. No significant reduction in viability was observed in any siRNA-treated group compared with the negative control (siNC) group (**Supplementary Figure S1**).

## 4 Discussion

In 2016, the World Health Organization (WHO) classified ZIKV infection as a Public Health Emergency of International Concern. ZIKV spreads through sexual contact, vertical transmission, and *Aedes* mosquito bites. Infection can lead to severe neurological disorders, such as GBS and meningoencephalitis in infected individuals and congenital microcephaly in infants born to infected mothers. To date, there are still no approved therapeutic drugs or preventive vaccines for ZIKV. The IFN-I system is the host’s core defense mechanism against viral infections: studies have proven that *Ifnar1*^−/−^ mice show a significantly increased susceptibility to lethal ZIKV infection (23), while exogenous IFN-β supplementation not only inhibits ZIKV replication in human vaginal/cervical epithelial cells but also mitigates ZIKV-induced neurotoxicity in brain organoids (14). However, among the hundreds of ISGs induced by IFN-I, which of them play key roles in the pathogenesis of ZIKV remains unclear.

ZIKV can directly invade the host’s nervous system and cause neural damage, and previous studies have reported that IFN-β can alleviate ZIKV-induced neural damage (24). To identify the key interferon effector genes mediating this neuroprotective effect, this study analyzed the gene expression profiles of ZIKV-infected brain organoids with and without IFN-β treatment, which identified 91 DEGs. Notably, 33% of the DEGs overlapped between the IFN-β-treated brain organoids and the PBMCs from naturally infected patients (**Figure 4A**), indicating that the ISG functions exhibit both conservation and tissue specificity between the immune and nervous systems. These shared ISGs include members previously reported to have ZIKV neuroprotective effects. For instance, Richard et al. confirmed that *RSAD2* (viperin) exerts a protective role by inhibiting ZIKV replication in the central nervous system (25). Subsequent GO and KEGG analyses showed that the pathways enriched in the IFN-β-treated brain organoids are mostly related to viral defense responses, consistent with the interferon signaling pathways activated during natural ZIKV infection. Nevertheless, the ISGs induced by natural infection did not fully overlap with those induced by IFN-β treatment (**Figure 4A**)—a finding consistent with previous research, as ZIKV can antagonize the host’s interferon signaling pathway via its encoded viral proteins to promote its own replication.

To further analyze the expression characteristics of the IFN-β-driven interferon effector genes at the single-cell level, this study examined the single-cell transcriptome of ZIKV-infected human moDCs, well-recognized ZIKV target cells. As expected, the ZIKV-infected moDCs pretreated with IFN-β showed higher ISG activity than the untreated group. Cell clusters were classified into ISG_high and ISG_low groups based on the ISG activity scores, and differential gene analysis was conducted using the FindMarkers function. The results revealed that the IFN-β-induced ISGs in moDCs differed from those in naturally infected PBMCs and ZIKV-infected brain organoids, suggesting significant heterogeneity in the ISG responses across different tissue sources and cell types (**Figure 4A**).

PBMCs are an ideal model for studying host responses to natural ZIKV infection. This study further analyzed the DEGs in the PBMCs from ZIKV-infected patients during the acute and convalescent phases, which identified 210 significant DEGs. GO and KEGG analyses indicated that these DEGs were mainly enriched in immune-related pathways and virus infection-related pathways [e.g., pathways associated with influenza A, hepatitis C, and coronavirus disease 2019 (COVID-19)], suggesting that ZIKV infection may activate signaling pathways common to other viral infections. In addition, enrichment of the pathways related to systemic lupus erythematosus (SLE) was observed (**Figure 3D**). The host mounts a robust yet self-limiting defense against ZIKV. This is evidenced by a transient upregulation of ISGs (e.g., *IRF7* and *SAT2*) during the acute phase, which resembles the interferon signature in SLE, but, unlike SLE, gradually fades during convalescence. This pattern aligns with the clinical observation that “ZIKV infection induces immune hyperactivation followed by viral clearance”.

By integrating multisystem transcriptomic data, this study systematically depicted the dynamic response characteristics of the ISGs in the immune and nervous systems during ZIKV infection. To screen for key ISGs regulating ZIKV infection across systems, Venn diagrams were used to identify the overlapping DEGs among three independent datasets (brain organoids, moDCs, and PBMCs), ultimately obtaining 22 candidate genes. These candidate ISGs are representative across multiple systems infected with ZIKV and have close molecular interactions, forming a core anti-ZIKV ISG network (**Figure 4B**). The anti-ZIKV functions of some of these genes have been confirmed: *IRF7*, a core transcription factor of the IFN-I signaling pathway, can further amplify the host’s antiviral response through positive feedback when upregulated; *IFITM1* and *IFITM3* restrict ZIKV entry into cells and subsequent replication by inhibiting fusion between the viral envelope and the host cell membrane; and *IFIH1* (MDA5), a PRR for RNA viruses, can specifically recognize ZIKV RNA and initiate IFN-I production. To evaluate the drug development potential of these candidate genes, the CMap database was used to predict potential compounds targeting these genes, with the top 20 scored compounds detailed in **Table 1**.

To verify the potential of the 22 candidate ISGs as biomarkers for ZIKV infection, a LASSO logistic regression model was used to screen the 11 potential diagnostic marker genes. The results showed that all 11 genes were significantly upregulated during the acute phase compared with the convalescent phase (**Figure 5**). Further validation via infection experiments in the A549 cell line confirmed that all 11 genes were upregulated after ZIKV infection, with *IFI27* and *IFI44* showing the most significant upregulation (notably, *IFI27* has been reported as a key ISG in dengue infection) (22). Among the 11 candidate genes, seven genes (i.e., *IFI44*, *IFI35*, *IFI27*, *IFIT3*, *OAS3*, *TNFSF10*, and *EPSTI1*) with no previous reports of association with ZIKV infection were selected for functional verification via siRNA-mediated gene knockdown. The results showed that the ZIKV RNA copy numbers in cells increased significantly after knockdown of *IFI35*, *IFI44*, or *OAS3* (**Figure 7**), indicating that these three ISGs are endogenous host restriction factors that inhibit ZIKV replication. Having established their functional role, we further evaluated the diagnostic potential of *IFI35*, *IFI44*, and *OAS3*. Receiver operating characteristic (ROC) curve analysis demonstrated that all three genes could effectively discriminate between acute and convalescent phases, with area under the curve (AUC) values of 0.885 for *IFI44*, 0.836 for *OAS3*, and 0.747 for *IFI35* (**Supplementary Figure S2**).

Therefore, this work builds upon existing reports in two key aspects: firstly, by providing direct loss-of-function evidence that establishes *IFI35*, *IFI44*, and *OAS3* as bona fide ZIKV restriction factors in a relevant human cell model, thereby providing crucial human cell validation for a role previously suggested for the murine homolog *Ifi44l* (26), and, secondly, by quantitatively defining their high diagnostic utility as biomarkers through ROC analysis, with *IFI44* emerging as a particularly strong candidate (AUC = 0.885).

This result confirms that the analytical strategy established in this study can effectively identify ISGs with anti-ZIKV potential in a standardized cell model. While the A549 cell line provided a robust and well-controlled system for the initial systematic screening of ISG functions, we acknowledge that this model does not fully represent the primary cellular tropism of ZIKV *in vivo*. Therefore, our findings position these ISGs, particularly *IFI35*, *IFI44*, and *OAS3*, as high-priority candidates for future investigation in more physiologically relevant models, such as neural organoids or primary immune cells, in order to further solidify their antiviral relevance and explore tissue-specific mechanisms.

## 5 Conclusion

The 2020 COVID-19 pandemic further drew the attention of the scientific community to the global public health threats posed by emerging viruses (27). The WHO has designated monkeypox outbreaks in multiple countries as a “Public Health Emergency of International Concern,” and the continuous spread of the highly pathogenic avian influenza A (H5N1) virus also poses a major threat to human and animal health (28). Single-stranded RNA viruses of the *Flavivirus* genus can cause severe human diseases, with their prevalence linked to insect vectors, urbanization, climate change, and global travel. Among these, ZIKV has become a key pathogenic virus of concern in this family due to its association with congenital microcephaly and fetal death. Currently, there are no approved drugs or vaccines for the prevention and treatment of ZIKV: antiviral strategies mainly target either the virus itself or the host factors, with the latter avoiding therapy failure caused by viral mutations. The IFN signaling pathway and related genes form the host’s first line of defense against ZIKV, providing key targets for host-directed therapy. Through comprehensive transcriptomic analysis, this study fills gaps in the understanding of the host interferon response mechanisms during ZIKV infection, and its research approach and conclusions can be extended to the field of flaviviruses. At the same time, by identifying key ISGs in infection, this study provides theoretical support for ZIKV prevention and treatment strategies and serves as a reference for related research.

## Data Availability

The original data presented in the study are publicly available. This data can be found in the NCBI Gene Expression Omnibus (GEO) repository under accession numbers GSE230571, GSE123818, and GSE129882.

## Supplementary material

Supplementary Figure 1 Assessment of cell viability after siRNA transfection. Cell viability was determined by the CellTiter-Glo assay and is expressed as a percentage relative to the siNC (scrambled negative control) group. Data are shown as mean ± SD (n=3). No statistically significant differences were observed (NS, P > 0.05; student’s t-test).

Supplementary Figure 2 **|** Receiver operating characteristic (ROC) curve analysis of candidate genes. The analysis was performed to evaluate the ability of indicated genes (IRF7, IFIT3, IFI35, IFI44, IFIH1, IFI27, IFITM1, TNFSF10, RSAD2, OAS3, EPSTI1) to discriminate between acute-phase and convalescent-phase patients of ZIKV infection. The Area Under the Curve (AUC) for each gene is indicated in the legend.
