# Supplementary figures and images for: Study on the spatiotemporal regulation of interferon-stimulated genes during Zika virus infection
Source: Front Immunol. 2026 Jan 7;16:1702266. doi: 10.3389/fimmu.2025.1702266 (PMC12819810; doi:10.3389/fimmu.2025.1702266)

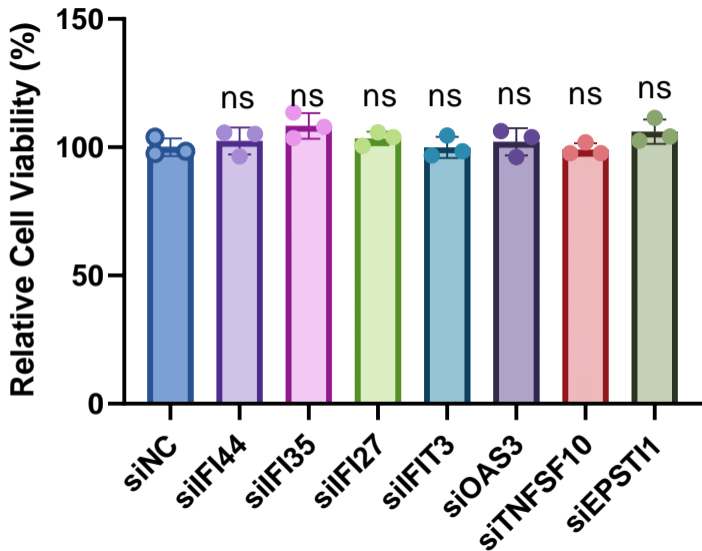

Supplement: Supplementary Figure 1 — Assessment of cell viability after siRNA transfection. Cell viability was determined by the CellTiter-Glo assay and is expressed as a percentage relative to the siNC (scrambled negative control) group. Data are shown as mean ± SD (n=3). No statistically significant differences were observed (NS, P > 0.05; student’s t-test). [file Image1.pdf]

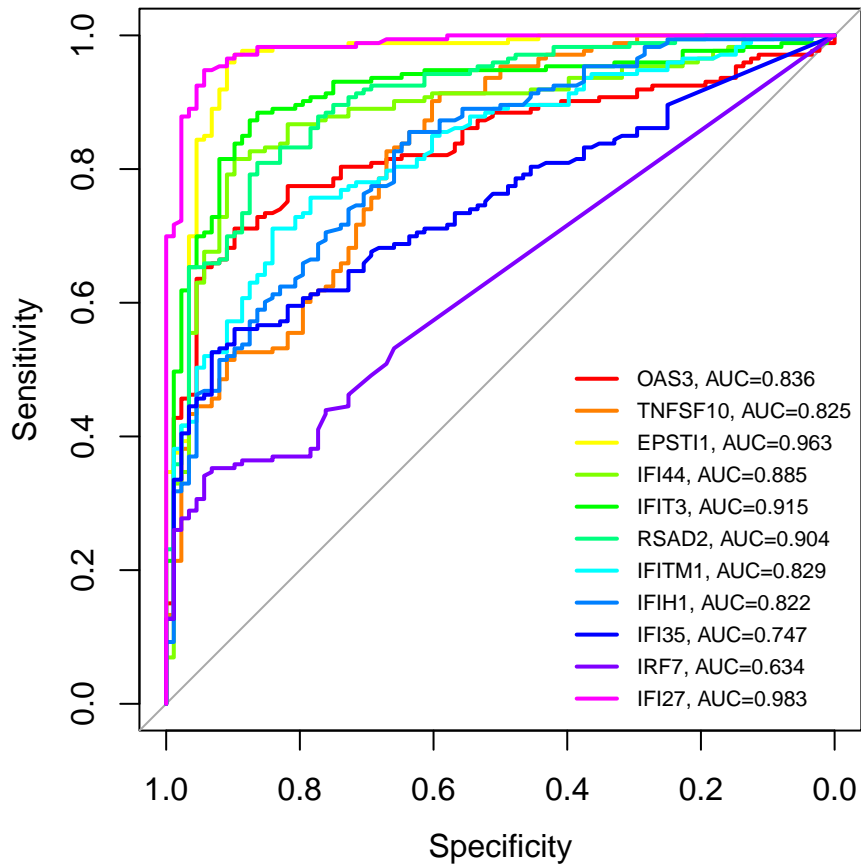

Supplement: Supplementary Figure 2 — | Receiver operating characteristic (ROC) curve analysis of candidate genes. The analysis was performed to evaluate the ability of indicated genes (IRF7, IFIT3, IFI35, IFI44, IFIH1, IFI27, IFITM1, TNFSF10, RSAD2, OAS3, EPSTI1) to discriminate between acute-phase and convalescent-phase patients of ZIKV infection. The Area Under the Curve (AUC) for each gene is indicated in the legend. [file Image2.pdf]
